# Thyroid hormone T3 induces Fyn modification and modulates palmitoyltransferase gene expression through αvβ3 integrin receptor in PC12 cells during hypoxia

**DOI:** 10.1515/tnsci-2022-0347

**Published:** 2024-08-07

**Authors:** Elisabed Kvergelidze, Tamar Barbakadze, Judit Bátor, Irine Kalandadze, David Mikeladze

**Affiliations:** Faculty of Natural Sciences and Medicine, Ilia State University, Tbilisi, 0162, Georgia; Laboratory of Biochemistry, Ivane Beritashvili Center of Experimental Biomedicine, Tbilisi, 0160, Georgia; Department of Medical Biology and Central Electron Microscopic Laboratory, Medical School, University of Pécs, Pécs, 7624, Hungary; Janos Szentagothai Research Centre, University of Pécs, Pécs, 7624, Hungary

**Keywords:** T3, hypoxia, Fyn, palmitoyltransferase, palmitoylation, JAK/STAT, SHP2, PC-12

## Abstract

Thyroid hormones (THs) are essential in neuronal and glial cell development and differentiation, synaptogenesis, and myelin sheath formation. In addition to nuclear receptors, TH acts through αvβ3-integrin on the plasma membrane, influencing transcriptional regulation of signaling proteins that, in turn, affect adhesion and survival of nerve cells in various neurologic disorders. TH exhibits protective properties during brain hypoxia; however, precise intracellular mechanisms responsible for the preventive effects of TH remain unclear. In this study, we investigated the impact of TH on integrin αvβ3-dependent downstream systems in normoxic and hypoxic conditions of pheochromocytoma PC12 cells. Our findings reveal that triiodothyronine (T3), acting through αvβ3-integrin, induces activation of the JAK2/STAT5 pathway and suppression of the SHP2 in hypoxic PC12 cells. This activation correlates with the downregulation of the expression palmitoyltransferase-ZDHHC2 and ZDHHC9 genes, leading to a subsequent decrease in palmitoylation and phosphorylation of Fyn tyrosine kinase. We propose that these changes may occur due to STAT5-dependent epigenetic silencing of the palmitoyltransferase gene, which in turn reduces palmitoylation/phosphorylation of Fyn with a subsequent increase in the survival of cells. In summary, our study provides the first evidence demonstrating the involvement of integrin-dependent JAK/STAT pathway, SHP2 suppression, and altered post-translational modification of Fyn in protective effects of T3 during hypoxia.

## 1 Introduction

Thyroid hormones (THs), specifically thyroxine (T4) and triiodothyronine (T3) are vital for maintaining the body’s homeostasis and functioning. They play essential roles in neuronal and glial cell development and differentiation, synaptogenesis, and myelin sheath formation [1]. Through genomic and non-genomic mechanisms, THs affect organs, including the brain. Genomic action involves binding to nuclear receptors, directly regulating gene expression [2]. The non-genomic action of TH is a rapid process involving the interaction of THs with various cellular components, including the cytoskeleton and mitochondria [3]. Besides these actions, TH binds to plasma membrane integrin αvβ3, changing the transcriptional regulation of signaling proteins in downstream pathways [4,5]. These interactions lead to alterations in adhesion, growth, and proliferation.

Twenty-four distinct integrin heterodimers are expressed in mammals due to the combinatorial association of 18α and 8β subunits. Extracellular matrix ligands bind to the α subunit and activate intracellular signaling events via the β subunit to integrate extracellular and intracellular events necessary for cell motility, migration, and invasion [6]. The intracellular structures formed by integrins and cytoskeletal proteins are “focal complexes.” The first steps of integrin signaling involve interactions with tyrosine kinases such as focal adhesion kinase (Fak), Src kinase, integrin-linked kinase, cytoskeletal proteins such as talin and kindlin, and scaffold molecules such as P130CRK-associated substrate (P130Cas) [7]. Besides, integrins regulate the dynamics of the neuronal actin cytoskeleton, ultimately enhancing neuronal cell viability through the Rac1/NADPH oxidase/cofilin-1 pathway [8].

Increasing evidence from clinical and preclinical studies reveals the critical roles of the non-receptor tyrosine kinase (nRTK) superfamily in the pathophysiology and therapy of cognitive disorders [9]. To date, several nRTK members from three nRTK subfamilies, i.e., the Src family kinase (SFK), the Janus tyrosine kinase (Jak), and the focal adhesion kinase (Fak) subfamilies, may connect to the intracellular, intranuclear, and synaptic signaling network linking chronic stress to depression- and anxiety-like behavior [10]. Emerging evidence shows that nRTK members from these three families are sensitive to stress [11]. Recent investigations have suggested that T3 decreases integrin αvβ3-dependent Fyn-kinase phosphorylation [8]. Fyn is a non-receptor tyrosine kinase of the Src family, abundant in the central nervous system. Fyn deficiency in mice leads to impaired hippocampal long-term potentiation, spatial learning deficits, and increased fear and audiogenic convulsion sensitivity in new generations [12]. Fyn activation can render neurons more susceptible to synaptotoxicity, while reduced activation has neuroprotective effects. However, excessive inhibition may impair cognitive function in humans. Thus, maintaining a delicate balance between Fyn activation and inhibition is crucial [13]. Overactivation of Fyn has been linked to brain pathogenesis induced by ischemia, potentially initiating apoptosis [14]. Besides Fyn, Jak tyrosine kinase plays a significant role in regulating the brain’s cognitive functions. Several studies show that JAK2/STAT5 signaling cascade is critical for neuropsychiatric disorders [15].

The activation of downstream signaling pathways within the cell is intricately regulated by post-translational modifications of upstream signaling proteins, including palmitoylation. Palmitoylation is a lipid modification of proteins involving the addition of palmitate residues to cysteine residues. This modification anchors proteins to subcellular membranes like the plasma membrane, allowing control over their membrane localization and cellular responses [16,17]. Palmitoylation is mediated by the protein palmitoyltransferase belonging to the DHHC (Asp-His-His-Cys motif) family, which transfers palmitoyl moiety to the palmitoylated protein [18,19]. Therefore, differential ZDHHC gene expression may influence protein translocations and play a significant role in the synaptic and metabolic activities of the brain. There are 23 distinct palmitoyltransferases identified in mammals, mainly located in the membrane of organelles, such as the endoplasmic reticulum, Golgi apparatus, and cell membrane [20]. In neurons, palmitoylation dynamically regulates the trafficking of proteins between the plasma membrane and subcellular structures like the Golgi apparatus, endoplasmic reticulum, and endosomes [21]. Palmitoylation plays a pivotal role in neural physiology, like neuroplasticity, and errors in palmitoylation/depalmitoylation can lead to brain pathology [22].

Hypoxia, a condition characterized by insufficient tissue-level oxygen, can result from reduced blood supply or decreased blood oxygen levels. It can manifest acutely or chronically, with varying tissue responses. Some tissues are highly resistant to hypoxia, while others are more vulnerable to brief exposures [23]. The impact of hypoxia on the brain depends on its type, duration, severity, and frequency. Hypoxia can trigger the release of inflammatory mediators like TNFα and IL1β, leading to local inflammation. It can also result in depolarization changes, oxidative stress, apoptosis, and neurodegeneration [24,25]. In several cell types, hypoxia selectively enhances integrin receptor expression [26]. In the brain, integrin activity changes during inflammation, hypoxia, and stress [27]. TH signaling has been implicated in hypoxic tissue remodeling after infarction, and T3 prevents remodeling of the postinfarcted tissue, decreasing secondary organ failure [28]. However, the precise intracellular mechanisms responsible for the protective effects of THs and the regulatory systems implicated in these processes remain unclear. Our hypothesis posits that non-receptor tyrosine kinases, associated with integrin activity and palmitoyltransferase gene activity in the cells, mediate the TH-dependent protective responses. In this study, we observed that T3, through integrin-αvβ3, triggers the JAK2/STAT5 pathway activation in PC12 cells. This activation is associated with integrin-dependent downregulation of the palmitoyltransferase-ZDHHC2 and ZDHHC9 gene expression and possibly the consequent reduction of palmitoylation of the tyrosine kinase Fyn. We propose that these changes may occur due to the STAT5-dependent epigenetic silencing of the palmitoyltransferase gene, which in turn reduces palmitoylation/phosphorylation of Fyn with a subsequent increase in the survival of cells [8]. In our interpretation, these molecular changes culminate in a reconfiguration of the actin cytoskeleton, ultimately enhancing neuronal cell viability [8].

## 2 Materials and methods

### 2.1 Cell line

Pheochromocytoma cells (PC-12, ATCC^®^ CRL-1721™) were cultured in a humidified atmosphere containing 5% CO_2_ at 37°C in a high-glucose Dulbecco’s modified Eagle’s medium supplemented with 10% heat-inactivated horse serum (HS), 5% fetal bovine serum (FBS), and 100 unit/mL penicillin as well as 50 µg/mL gentamicin sulfate. To induce differentiation, PC-12 cells (5 × 10^6^ cells per sample) were incubated in low serum-containing DMEM (1% HS and 1% FBS) supplemented with 100 ng/mL nerve growth factor (NGF) for 5 days. The cells were scored as differentiated if one or more neurites were longer than the cell body diameter. Cell viability and number were determined with trypan blue dye (Bio‐Rad, cat no. 145‐0013) using a cell counter (TC 20TM; Bio‐Rad, USA). Differentiated cells were incubated with 10 nM T3 and αvβ3 blocking antibody (αvβ3-Ab, 1 µg/ml; sc-7312, Santa Cruz Biotechnology). The experiments were performed under hypoxic conditions for 1 h. Hypoxic conditions (0–1% oxygen) were maintained using nitrogen gas in a BioSpherix C-Chamber placed in a CO_2_ incubator and controlled by a ProOx Model P110 controller (BioSpherix, USA).

### 2.2 Cell fractionation for electrophoresis and western blotting

After 1 h exposure to hypoxia, PC-12 cells were detached from the cell culture flasks using 0.025% trypsin/EDTA containing phosphate-buffered saline (PBS) [8]. Briefly, trypsin inactivation of detached cells was performed using aprotinin-containing PBS (1 µg/mL). Cells were washed twice with PBS and pelleted by centrifugation at 300 × *g*. Incubated PC-12 cells were lysed using a lysis buffer. For the palmitoylation detection, 1% IGEPAL CA‐630, 50 mM Tris‐HCl, 150 mM NaCl, 10% glycerol, 50 mM *N*-ethylmaleimide, PMSF, and PI Cocktail III; pH 7.5 containing lysis buffer (LB) were used and passed through a 25Ga needle 10 times using a 1 mL syringe. For the JAK/STAT pathway protein analysis, cells were lysed with lysis buffer provided by RayBiothec. After cell lysis, the nuclei and intact cells were sedimented at 720 × *g* for 5 min, and the supernatant was subjected to the next steps.

### 2.3 Palmitoylated Fyn and p-Fyn detection

For specific detection of palmitoylated Fyn and palmitoylated p-Fyn, samples (0.3 mg) were immunoprecipitated with anti-Fyn (sc-434; Santa Cruz Biotechnology, USA) or anti-p-Fyn (sc-377555; Santa Cruz Biotechnology, USA) primary antibody and incubated overnight at 4°C with gentle shaking. The next day, A/G agarose was added to each sample (sc-2001; Santa Cruz Biotechnology, USA) and incubated for 4 h at 4°C with gentle shaking. After incubation, samples were washed with PBS buffer three times by centrifugation at 300 × *g* for 2 min; the protein/primary antibody/A/G-agarose precipitate was subsequently used to determine the palmitoylation level of target proteins.

Palmitoylated Fyn (palm-Fyn) and palmitoylated and phosphorylated Fyn (palm-p-Fyn) kinases were detected in the Fyn-anti-Fyn/A-G-agarose and p-Fyn/anti-p-Fyn/A-G-agarose immunoprecipitate according to Brigidi and Bamji [29] and Goloshvili et al. [17]. Briefly, A/G-agarose bound samples were incubated with 10 mM *N*-ethylmaleimide (NEM) containing lysis buffer (LB + NEM buffer) and incubated for 10 min on ice. Then, the suspension was centrifuged at 500 × *g* at 4°C, and a Stringent Buffer was added to the samples, centrifuged very quickly, and the supernatant was removed. The residue was washed three times with LB pH 7.2 (LB_7.2_) buffer (washing includes 1-min centrifugation at 500 × *g* at 4°C). Then, newly prepared hydroxylamine (1 M HAM) containing LB_7.2_ + 10 mM NEM was added to the residue as a potent reducing agent for removing palmitate groups from cysteine residues in Fyn and p-Fyn kinase molecules. Samples were incubated for 1 h at room temperature with slow shaking. After incubation, the supernatant was removed, and each sample was washed once on ice with lysis buffer (LB, pH 6.2 PMSF/PI (LB_6.2_). For further biotinylation, thiol groups on free cysteine residues, Biotin-BMCC buffer (Sigma Aldrich, cat no. B9181) (working concentration: 0.5–5 µM) were added to the A/G-agarose complex of Fyn and p-Fyn of each sample and incubated for 1 h at 4°C with gentle shaking. After incubation, each sample was washed once on ice with LB_6.2_ PMSF/PI. Subsequently, samples were washed three times with LB_7.2_ + PMSF/PI. All samples were placed on ice during the above washing process. Next, 2× sodium dodecyl sulfate (SDS) assay buffer without mercaptoethanol (5% SDS, 5% glycerol, 125 mM Tris‐HCl, pH 6.8, and 0.01% bromophenol blue) was added to the samples with freshly prepared dithiothreitol. The content of biotin-BMCC bound to palm-Fyn and palm-p-Fyn was determined by electrophoresis and western blotting methods using immunolabeled streptavidin-HPR and visualized with an enhanced chemiluminescence kit (cat no. sc2048, ECL, Santa Cruz Biotechnology) and was analyzed using the Image J (1.53 k, National Institute of Health, USA).

### 2.4 Detection of the relative levels of phosphorylation of JAK/STAT pathway proteins

The relative levels of JAK/STAT pathway protein phosphorylation were detected using the RayBio^®^ JAK/STAT Pathway Phosphorylation Array Kit (Cat# AAH-JAKSTAT-1-8; RayBiotech) according to the manufacturer’s manual. After quantification using the BCA kit, 100 μg of protein was loaded onto a RayBio Human Signaling Pathway Antibody Array membrane, and the steps mentioned in the manual were followed. In the end, immunolabeled bands on the membrane were visualized using enhanced chemiluminescence (sc-2048; Santa Cruz Biotechnology, USA) and analyzed by Image J (1.53 k, National Institute of Health, USA). The internal control signals of each protein array chip were used for standardization. A *t*-test was used for the difference analysis, and fold changes ≥1.5 times were considered significant.

### 2.5 RNA extraction with Trizol reagent

Trizol reagent is used to isolate RNA. About 1 ml Trizol was added to each sample and incubated for 5 min. Later, 200 µl/l chloroform was added, incubated for 15 min on ice, and centrifuged at 12,000 × *g* for 15 min at 4°C. RNA is precipitated from the aqueous layer with isopropanol and isolated according to Invitrogen’s user guide (TRIzol Reagent User Guide (Pub.No. MAN0001271 C.0). The amount and purity of the obtained RNA was determined spectrophotometrically using nanodrop (DeNovix DS-11 Spectrophotometer, DeNovix Inc., USA). The ratio *A*_260_/*A*_280_ should be >1.8. The RNA residue was dissolved in DEPC water and stored at −70°C.

### 2.6 cDNA synthesis using the RevertAid RT Kit

According to the protocol, the RevertAid RT Kit was used for the cDNA synthesis (cat no. K1691, Thermo Scientific) specified by the manufacturer. One microliter of Random hexamer primer was added to 2 μg RNA-containing samples and filled until 12 μL with nuclease-free water. This step was followed by the addition of 4 μL of 5× Reaction buffer, 1 μL RiboLock, 2 μL 10 mM DNTP, and 2 μL RevertAid. Finally, 20 μL of the mixture was incubated for 5 min at 25°C and 60 min at 42°C. The reaction was terminated by heating at 70°C for 5 min. The reverse transcription reaction product was stored at −70°C for real-time PCR experiments.

### 2.7 PCR amplification of first strand cDNA

The relative level of palmitoyl transferase genes 2, 3, 8, 9, and 16 (genes: Zdhh2, Zdhh3, Zdhh8, Zdhh9, Zdhh16, and housekeeping gene: Hprt1 – hypoxanthine phosporibosyl transferase) expression was analyzed using RT-RCR. Appropriate primers were selected for PCR by Integrate DNA Technology (https://eu.idtdna.com/scitools/Applications/RealTimePCR/Default.aspx) and using Primer-BLAST (Table 1) (https://www.ncbi.nlm.nih.gov/tools/primer-blast/index.cgi) and were ordered to Eurofins Genomics, Ebersberg, Germany (Table 1).

**Table 1:** Primers for real-time PCR analyzing the genes: Zdhh2, Zdhh3, Zdhh8, Zdhh9, Zdhh16, and housekeeping gene Hprt1

Ref seq gene	Accession number	Forward primer (5′–3′)	Reverse primer (5′–3′)	Ampl. size
Hprt1	NM_012583.2	GTTCTGTCATGTCGACCCTC	AACACCTTTTCCAAATCTTCAGC	115
Zdhhc2	NM_145096	TGTATGCGGCTGGAAGATG	AGCTGATGAACACCACAGG	95
Zdhhc3	NM_001039014	ATGCCAGTATGGACAGAATAGC	GGCTGGAGGTATTCTGGTTTC	86
Zdhhc8	NM_001039021	TCAAACCCGCCAAGTACATC	ACAGCTCTTGTCAACCACG	103
Zdhhc9	NM_001039016	GATTTTCAAGCTCAGCCTCTG	TTTCTCCCACTTCCTTGTCAC	120
Zdhhc16	NM_001039346	TGAAACTTCTATGCGCCAGG	GAGCAGAGGTGGGCATC	120

Structured using Integrate DNA Technology (https://eu.idtdna.com/scitools/Applications/RealTimePCR/Default.aspx) and using Primer-BLAST. Hprt1: hypoxanthine phosphoribosyl transferase.

**Table 2:** RT-qPCR analysis of genes ZDHHC2, ZDHHC3, ZDHHC8, ZDHHC9, and ZDHHC16 showing the effect of 10 nM T3 and αvβ3-Ab integrin (µg/ml) in differentiated PC-12 cells exposed to 1 h hypoxia

	ZDHHC2 (2^−^(∆∆Ct)^)	ZDHHC3 (2^−^(∆∆Ct)^)	ZDHHC8 (2^−^(∆∆Ct)^)	ZDHHC9 (2^−^(∆∆Ct)^)	ZDHHC16 (2^−^(∆∆Ct)^)
Control	3.73	4.90	1.00	11.06	3.55
αvβ3-Ab	4.13	7.26	5.01	13.04	1.10
T3	1.92	5.02	0.75	6.97	4.82
T3+αvβ3-Ab	2.91	4.63	1.32	11.51	6.39

The results are expressed as 2^−^(∆∆Ct)^ after normalization on Hprt1 (hypoxanthine phosphoribosyl transferase) housekeeping gene. Results represent the mean ± SEM of duplicate samples from three independent experiments.

Sybr Green PCR master mix (cat no. 4309155; Applied Biosystems) was used in RT-PCR experiments. The primers were diluted according to the manufacture protocol to a final concentration of 10 pmol/l. About 2 μL of the first strand cDNA synthesis reaction product of each sample was used as a template, followed by the addition of the 1 μL of forward primer (5′–3′), 1 μL of reverse primer (5′-3′), 21 μL of DNase- and RNase-free water, and 25 DNase- and RNase-free water 2X SYBR green Master Mix for subsequent PCR in 50 μL of total volume. The final mixture of all samples was subjected to PCR using the recommended thermal cycling conditions outlined as follows:

**Table:** 

Step	Temperature (°C)	Time	Number of cycles
Initial denaturation	95	1–3 min	1
Denaturation	95	30 s	
Annealing	Tm-5	30 s	40
Extension	72	1 min/kb	
Final extension	72	5–15 min	1

### 2.8 Statistical analysis

Statistical analysis was performed using one-way ANOVA with *post-hoc* Tukey to compare multiple treatments. A value of *p* < 0.05 was considered as statistically significant.

## 3 Results

**Figure 1:**
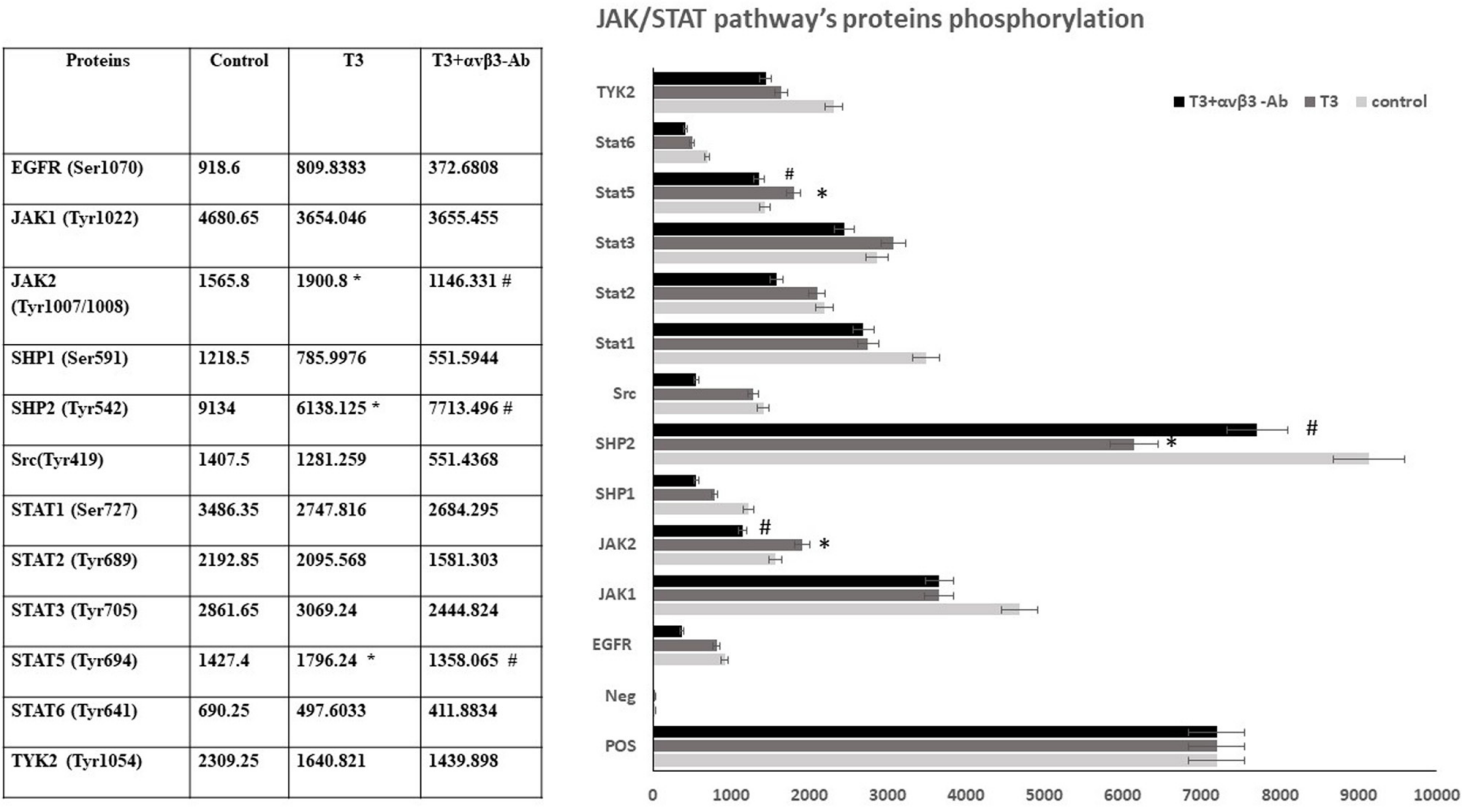
JAK/STAT pathway’s protein phosphorylation in differentiated PC-12 cells in hypoxia. Densitometric analysis (arbitrary units of spot signal densities normalized to the positive control signals) showing the effect of 10 nM T3 and αvβ3-Ab integrin (µg/ml). Results represent the mean ± SEM of duplicate samples from two independent experiments. A significant level is defined as *p < 0.05 vs control and #*p* < 0.05 to T3.

To elucidate the downstream regulatory systems involved in the effects of T3-dependent integrin activation during 1-h hypoxia, we analyzed the qualitative protein phosphorylation profile of the JAK/STAT pathway. Of the 12 phosphorylated proteins, the most significant changes were observed: JAK1 and JAK2, SHP1 and SHP2, STAT1, STAT3 and STAT5, and non-receptor tyrosine kinase – TYK2. Our results have shown that the phosphorylation of JAK1, SHP1, SHP2, and STAT1 decreased. In contrast, the phosphorylation of JAK2, STAT3, and STAT5 increased, and only the change in JAK2, STAT5, and SHP2 phosphorylation returned to control levels in the presence of an anti-integrin antibody (Figure 1). Phosphorylation of other proteins was not reversed in the presence of anti-integrin antibodies, indicating that, in these cases, the change in phosphorylation occurs independently of integrin. Therefore, only the integrin-dependent JAK2/STAT5 pathway is activated during hypoxia under the influence of T3.

**Figure 2:**
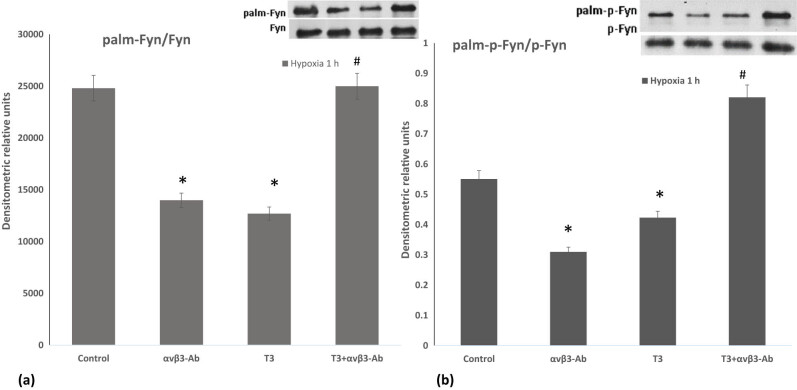
The action of T3 on the phosphorylation and palmitoylation of Fyn A. The ratio of palmitoylated and non-palmitoylated Fyn and p-Fyn forms. The palm-Fyn/Fyn ratio in differentiated PC-12 cells exposed to 10 nM T3 and µg/ml αvβ3-Ab integrin during 1 h of hypoxia. (a) Immunoblotting image of palm-Fyn and total Fyn; the palm-p-Fyn/p-Fyn ratio in differentiated PC-12 cells exposed to 10 nM T3 and αvβ3-Ab integrin (µg/ml) during 1 h of hypoxia. (b) Immunoblotting image of palm-p-Fyn and total p-Fyn; plot of the ratio of palm-p-Fyn to total p-Fyn. Plot of the ratio of palm-Fyn to total Fyn. Results represent the mean ± SEM of duplicate samples from two independent experiments. A significant level was defined as *p < 0.05 vs control and #*p* < 0.05 to T3.

Tyrosine kinase Fyn is involved in the phosphorylation and assembly/remodeling of the postsynaptic complexes in response to hypoxia/ischemia in the brain and Fyn coordinates with other non-receptor tyrosine kinases [30]. Fyn enhances Jak2-mediated phosphorylation [31], and both Fyn and Jak2 are required for ROS-dependent activation of redox-sensitive downstream systems [32]. Thus, the activity of Fyn kinase is closely related to the Jak2 pathway, in which post-translational modification could play a significant role. Our recent studies indicate that T3 reduces the p-Fyn/Fyn ratio, regulating actin cytoskeleton dynamics through αvβ3-integrin-mediated Fyn dephosphorylation [8] and by switching on anti-apoptotic pathways [33]. Considering that besides phosphorylation, the activity and localization of Fyn are regulated by palmitoylation [34], next, we investigated the αvβ3 integrin-mediated effects of T3 on Fyn palmitoylation and the phosphorylation of palmitoylated Fyn (at Thr12) during 1-h hypoxia. Our experiments revealed that T3 reduces Fyn palmitoylation, and this effect is reversed by αvβ3 integrin antibody (Figure 2). Additionally, T3 decreases the phosphorylation of palmitoylated Fyn, and inhibition of αvβ3-integrin by antibody significantly increases palmitoylated Fyn phosphorylation. Notably, inhibiting αvβ3 integrin without T3 reduces the phosphorylation of palmitoylated Fyn. These findings indicate that T3 activates Fyn phosphorylation, depending on protein palmitoylation.

**Figure 3:**
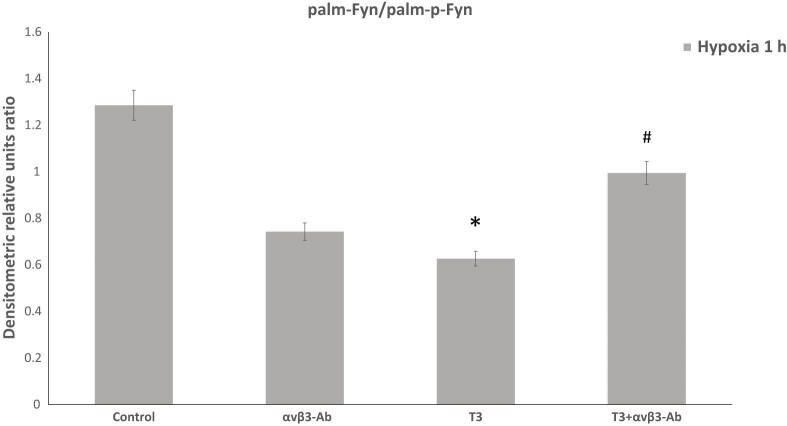
TH supplementation of hypoxic cells increases the proportion of phosphorylated to non-phosphorylated forms of palmitoylated Fyn kinase in differentiated PC-12 cells exposed to 10 nM T3 and αvβ3-Ab (µg/ml) integrin during 1 h hypoxia. Results represent the mean ± SEM of duplicate samples from two independent experiments. A significant level was defined as *p < 0.05. vs control and #*p* < 0.05. to T3.

Analyzing the ratio of phosphorylated to non-phosphorylated forms of palmitoylated Fyn in PC-12 cells revealed a significant effect of T3 and αvβ3 integrin. TH supplementation of hypoxic cells increases the proportion of phosphorylated to non-phosphorylated forms of palmitoylated Fyn kinase compared to controls, and αvβ3 integrin inhibition further enhances this ratio. However, declining the activity of αvβ3 integrin without T3 did not alter this ratio compared to control cells (Figure 3).

Based on these results, it can be inferred that in hypoxic PC-12 cells, crosstalk exists between the T3-mediated αvβ3 integrin-dependent Fak/STAT3 pathway and other nRTK-dependent pathways. This interplay contributes to complex cellular processes that mutually modulate each other [35].

Palmitoyltransferases are typically located within intracellular structures. The levels of these enzymes in the cells depend on the activity of the genes encoding these enzymes [36]. Thus, next, we determine the expression of genes involved in palmitoylation through quantitative analysis of mRNA. Specifically, we analyzed the expression of palmitoyltransferase genes 2, 3, 8, 9, and 16 owing to their critical roles in the nervous system [21,22]. The expression levels of ZDHHC2, 3, 8, 9, and 16 genes during 1-h hypoxia are shown in Table 2.

**Figure 4:**
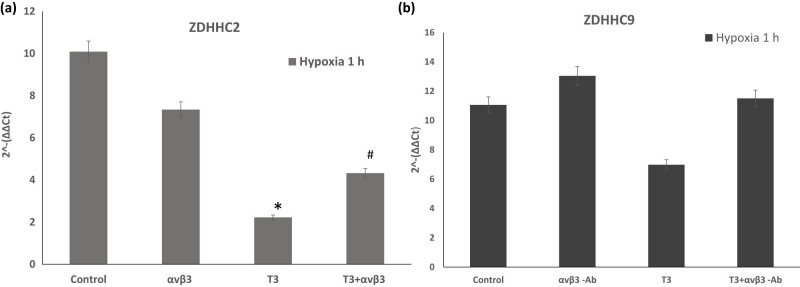
The palmitoyltransferase gene expression levels of (a) ZDHHC2 and (b) ZDHHC9It were determined using the RT-PCR method in differentiated PC-12 cells exposed to 10 nM T3 and αvβ3-Ab integrin (µg/ml) during 1 h hypoxia. Results represent the mean ± SEM of duplicate samples from three independent experiments. A significant level was defined as *p < 0.05 vs control and #*p* < 0.05 to T3.

We found that T3 does not alter the expression of ZDHHC3 and ZDHHC8 in hypoxia. However, when αvβ3 integrin was blocked, a significant increase in the expression of these genes was observed. Subsequently, we examined the expression of ZDHHC2 because there is some evidence that the activity of ZDHHC2 is associated with the palmitoylation of cytoskeleton-associated protein-4 (CKAP4), thereby suppressing cell proliferative activity in tumorigenesis [37]. T3 significantly reduces the expression of ZDHHC2, which is explicitly mediated by αvβ3 integrins (Figure 4a). Based on our results, we can assume that T3 does not affect the expression of ZDHHC3 and ZDHHC8 under hypoxic conditions. Still, it significantly reduces the expression of the ZDHHC2 gene, and this effect is partially resolved by blocking αvβ3 integrin. These data suggest that T3, in hypoxia, does not affect the expression of ZDHHC3 and ZDHHC8; however, it significantly reduces the expression of the ZDHHC2 gene.

Because mutation of ZDHHC9 palmitoyltransferase leads to the impairment of various cognitive functions and the development of epilepsy [38], and ZDHHC16 palmitoyltransferase plays an essential role in activating the early response to DNA damage [39], next, we analyzed the expression of these two genes. We found that T3 significantly reduces ZDHHC9 expression in hypoxic cells, and this effect was reversed in the presence of αvβ3 integrin-blocking antibody (Figure 4b).

Next, the expression of ZDHHC16 was determined. There is some evidence that this isozyme plays a significant role in the embryogenesis of the nervous system [40]. Besides, the importance of ZDHHC16 in early response to DNA damage has been suggested [39]. We found that T3 non-specifically increases the expression of ZDHHC16, but this effect is enhanced in combination with αvβ3 integrin blocking antibody. Based on the obtained data, it can be suggested that in contrast to the ZDHHC16 gene, T3 regulates the expression of ZDHHC9 through αvβ3 integrin activation. ZDHHC16 gene expression is also altered, although, unlike ZDHHC9, its expression is not αvβ3 integrin-specific and other T3-dependent mechanisms are involved in the expression of this gene.

## 4 Discussion

Accumulating evidence suggests that TH plays a critical role in the response of various tissues to ischemic/hypoxic insults. TH signaling has been implicated in ROS-dependent damage after infarction in hypoxic tissue, preventing post-infarcted remodeling [28,41]. These effects of THs may be due to genomic and non-genomic actions. The classical genomic action of T3 is mediated by high-affinity nuclear receptors that directly regulate gene expression. In contrast, the non-genomic effects of THs occur rapidly and are unaffected by transcription and protein synthesis inhibitors [3]. The non-nuclear actions of THs involve multiple physiological processes in many different cell types and are thought to be mediated by various intracellular regulatory systems. Sites of non-genomic action are localized to the plasma membrane, cytoplasm, cytoskeleton, and sub-cellular organelles. Recent advances have identified the plasma membrane integrin αvβ3 as a high-affinity receptor for T3 [42]. This research adds a new direction to THs’ actions because they promote the discovery of new mechanisms in cell growth and proliferation.

Integrin αvβ3 is a heterodimer found on the surfaces of cells that has an essential role in maintaining cell structure and signal transduction. Several small molecules, like resveratrol, non-peptide hormones like steroid hormones [43], and THs [44] have specific binding sites on integrin αvβ3 [35]. These ligands induce signal transduction and sequentially stimulate the biological activities of cells [42,44]. Focal adhesion kinase (Fak), a non-receptor tyrosine kinase, controls the activity of integrin αvβ3 and promotes cell migration and invasion [45].

Activation of proliferative genes via the integrin αvβ3/Fak pathway is one of the signaling actions of THs [46]. Several studies suggested that various non-receptor tyrosine kinases, including the Fak, can induce JAK/STAT cascade activation; however, the precise mechanisms involved in the phosphorylation and activation of JAK are unknown [47,48]. Our analysis of different non-receptor tyrosine kinases has shown T3-dependent phosphorylation of the JAK2/STAT5, suggesting an activation of this cascade system. Proper functioning of the JAK2/STAT5 signaling pathway relies on crosstalk with other signaling pathways, which leads to normal biological performance [49]. Considering that hypoxia itself may compensatory activate this signaling cascade [50,51], it may be concluded that the additional action of T3 can significantly increase the oxidative stress-induced responses. Simultaneously, T3 decreases SHP-2 protein phosphorylation during hypoxia, which affects the Fak kinase activation [52]. T3-induced effect is abolished under the action of integrin αvβ3 inhibitory antibody. Recent studies indicate that SHP2 has a crucial role in neurodegenerative brain diseases, and its suppression may be neuroprotective [53].

The JAK2-STAT5 pathway is essential for cellular development and survival [54]. Increased JAK2 activity increases BDNF expression and inhibits the apoptosis of neurons in chronic mild stress [55,56]. Moreover, hypoxia-induced JAK2/STAT5 activation could prevent neuronal apoptosis after ischemic injury [57]. Numerous studies have shown that STAT5 can also function as a transcriptional repressor by recruiting demethylating or deacetylating epigenetic modifiers in specific gene loci [58,59]. Lysine-specific histone demethylase 1A (LSD1) and HDAC3 also exert transcriptional regulation of STAT5 targets and facilitate specific gene repression by either deacetylation or histone demethylation [60]. These findings indicate that STAT5 may be involved in the epigenetic regulation of gene expression in physiological and pathological brain processes [61].

We found that Fyn kinase, a Src family member of tyrosine kinase, can also participate in the T3-dependent action in hypoxic cells. Fyn palmitoylation is essential for its localization to the membrane, and aberrant modification of this enzyme contributes to the pathophysiology of neurodegenerative diseases [62]. Our experiments have shown that T3 reduces Fyn palmitoylation and decreases its subsequent protein phosphorylation (at Thr12). Our recent studies indicate that a decline in the p-Fyn/Fyn ratio regulates actin cytoskeleton dynamics through αvβ3-integrin-mediated Fyn dephosphorylation [8]. These findings align with the observations that phosphorylation at the 12th threonine residue modulates Fyn kinase activity [63,64], and by reducing Fyn kinase, phosphorylation switches on anti-apoptotic machinery in neurons [33].

Elevated expression of palmitoylating enzymes [65] and integrins [66] was found in neurons. In this light, we analyzed the expression of five genes, which are highly distributed in nervous tissue. Our results show that T3 downregulates ZDHHC2 and ZDHHC9 gene expression in an integrin-specific manner. In contrast, the expression of other palmitoyltransferase genes, such as ZDHHC3, ZDHHC8, and ZDHHC16, changes slightly, or their differences are not integrin-specific. Thus, we can conclude that the TH T3, through the integrin αvβ3/JAK2/STAT5 pathway, suppresses the expression of the palmitoyltransferase genes ZDHHC2 and ZDHHC9, which is likely due to the inclusion of epigenetic silencing [64]. Notably, the DHHC family of protein palmitoyltransferases is epigenetically regulated through DNA methylation [67], which can be modulated by STAT5. It seems that these changes are a preventive effect protecting the brain from high-frequency stimulation and fear reactions [51].

**Figure 5:**
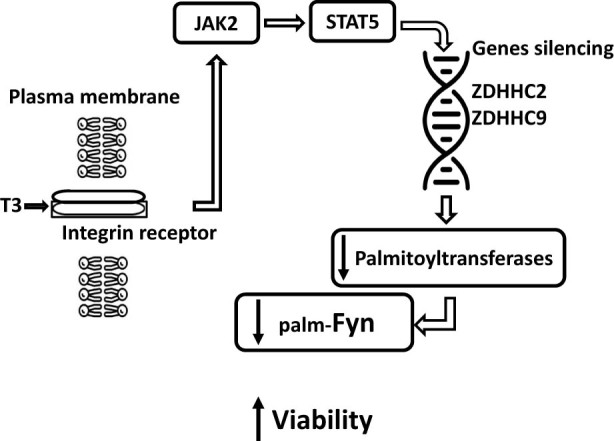
Hypothetical scheme of the beneficial effect of T3 on differentiated PC-12 cells during hypoxia.

In conclusion, our observations are the first to demonstrate the involvement of the integrin-dependent JAK2/STAT5 cascade and possible modulation of Fyn palmitoylation in the T3-mediated effects during 1-h hypoxia. This pathway and nRTKs are activated through integrin-αvβ3 and likely compensate for ROS-induced damage. In this case, a decrease in the expression of the ZDHHC2 and ZDHHC9 genes is observed, associated with changes in palmitoylation and redistribution of several proteins, including the protein kinase Fyn, that are beneficial for maintaining cell viability during hypoxia. These changes may occur due to the STAT5-dependent possible induction epigenetic silencing of the palmitoyltransferase gene, which in turn reduces palmitoylation/phosphorylation of Fyn with a subsequent increase in the survival of neurons [8] (Figure 5). These findings highlight the importance of further research using different research models to explore potential therapeutic interventions and their consequences.

## Supplementary Material

Supplementary Figure
