# Supplementary figures and images for: Thyroid hormone T3 induces Fyn modification and modulates palmitoyltransferase gene expression through αvβ3 integrin receptor in PC12 cells during hypoxia
Source: Transl Neurosci. 2024 Aug 7;15(1):20220347. doi: 10.1515/tnsci-2022-0347 (PMC11306964; doi:10.1515/tnsci-2022-0347)

Supplementary material

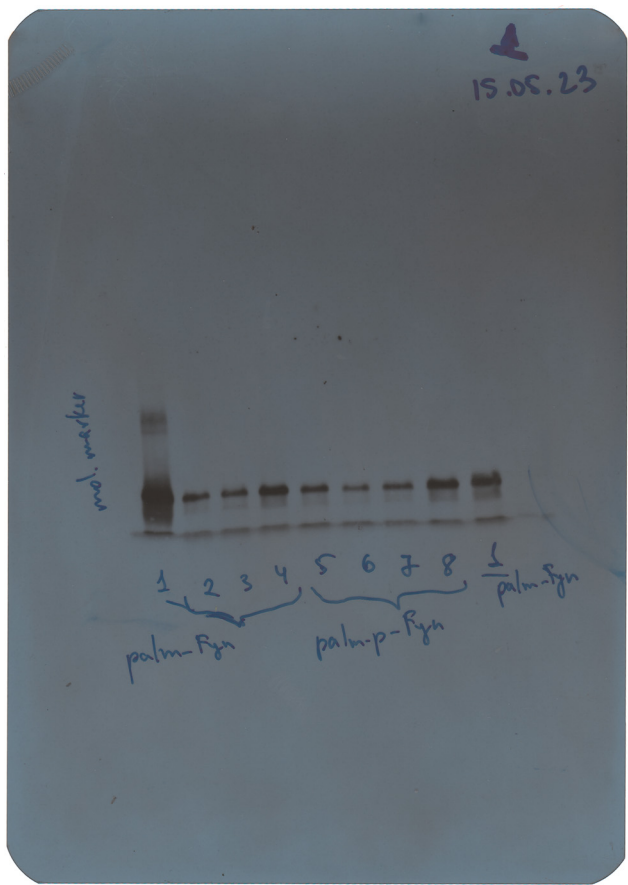

Blot 1: Control.

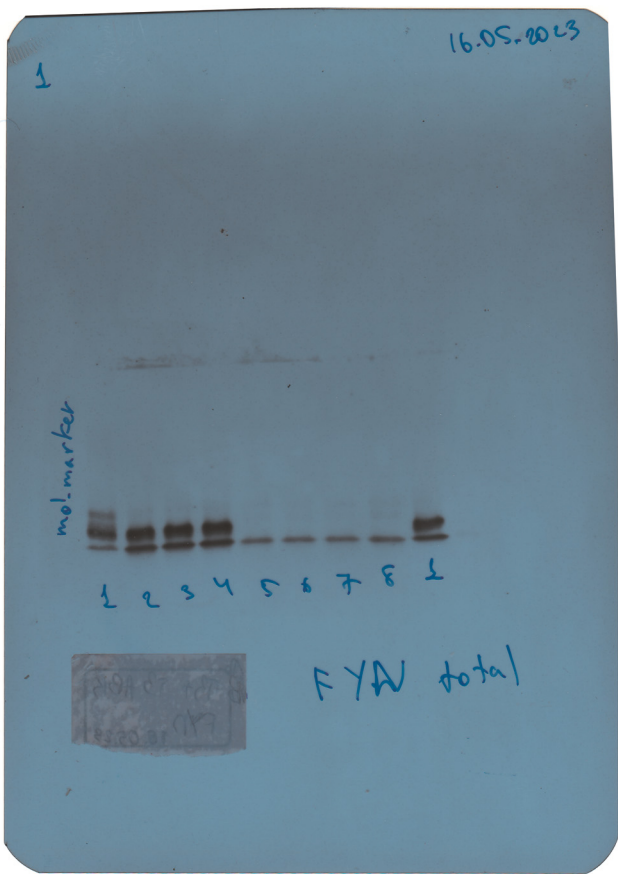

Blot 2:  $\alpha v\beta 3$ -Ab.

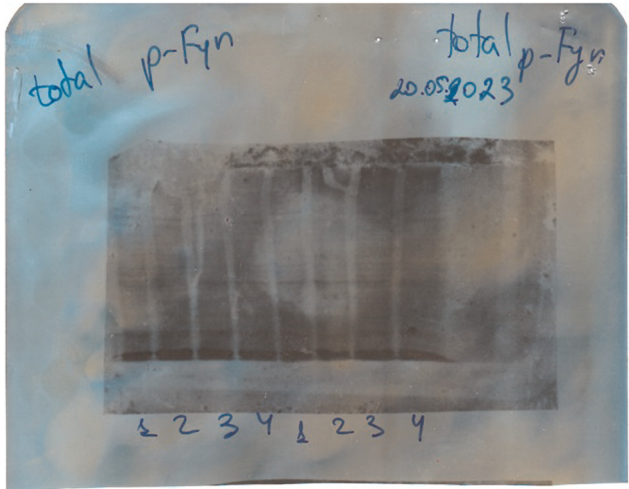

Blot 3: T3.

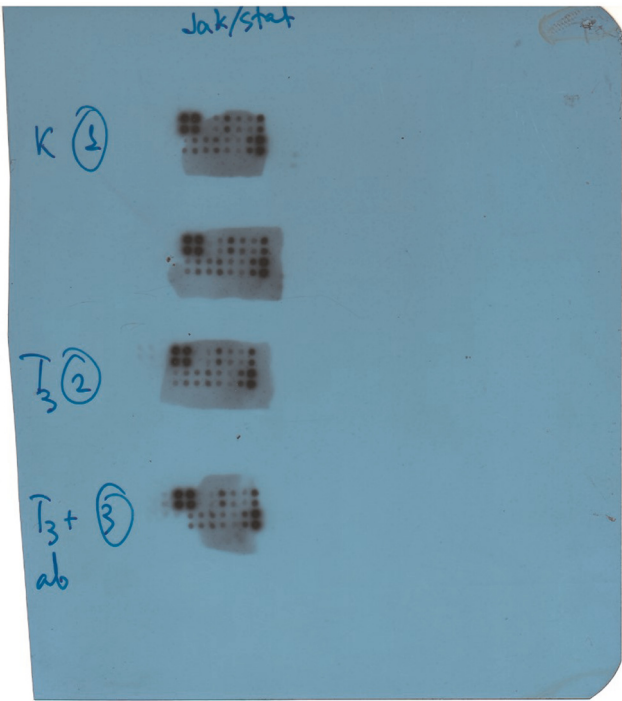

Blot 4: T3+αvβ3-Ab.

Supplement: Supplementary Figure [file tnsci-2022-0347-sm.pdf]
